# Recommended procedures for the management of early childhood caries lesions – a scoping review by the Children Experiencing Dental Anxiety: Collaboration on Research and Education (CEDACORE)

**DOI:** 10.1186/s12903-020-01067-w

**Published:** 2020-03-17

**Authors:** Patrícia Corrêa-Faria, Karolline Alves Viana, Daniela Prócida Raggio, Marie Therese Hosey, Luciane Rezende Costa

**Affiliations:** 1 Dentistry Graduate Program, School of Dentistry, Universidade Federal de Goiás, Praça Universitária, Goiânia, Goiás 74605220 Brazil; 2 Graduate Program in Dental Sciences, School of Dentistry, Universidade de São Paulo, Av Lineu Prestes, 2227, São Paulo, 05508-000 Brazil; 3 Head of Paediatric Dentistry, Faculty of Dentistry, Oral & Craniofacial Sciences, King’s College London, London, UK

**Keywords:** Dental caries, Child, preschool, Dental care, Guideline

## Abstract

**Background:**

Early childhood caries (ECC) affects millions of children up to 6 years old. Its treatment positively impacts the quality of life of children and their families. However, there is no consensus on how to treat ECC. Thus, we performed a scoping review to identify the recommended procedures for the management of ECC lesions.

**Methods:**

A search was performed in PubMed, Scopus, The Cochrane Library, The International Guideline Library and pediatric dentistry associations around the world were contacted by email for unpublished search documents. ECC guidelines/guidance/policies were considered eligible regardless of language and publication date.

**Results:**

From a total of 828 references, 52 full-text articles were assessed for eligibility and 22 included in the scoping review. We found different procedures recommendations for the management of ECC lesions. For incipient lesions, minimally invasive methods such as professional fluoride and cariostatic (silver diamine) applications, as well as surveillance were recommended. If restoration was required, the recommended materials were glass ionomer cement, composite resin, amalgam and stainless-steel crown. Interim restorations and Atraumatic Restorative Treatment (ART) were also recommended. Extractions have been suggested for teeth with lesions with pulpal involvement, depending on the child’s behaviour and other clinical conditions.

**Conclusions:**

Non-operative procedures, restorative and extraction were recommended for the management of ECC, depending on the extent of the lesions. There is no difference between different management guidelines/guidance/policies for ECC lesions.

## Background

Millions of children under 6 years of age have early childhood caries (ECC) globally [1–3]. This condition is a multifactorial and dynamic disease characterized by “the presence of one or more decayed (non-cavitated or cavitated lesions), missing (due to caries), or filled surfaces, in any primary tooth of a child under age six” [2, 4]. The prevalence of this condition is closely related to the child’s age. Thus, while the mean ECC prevalence in children at 1 year of age was 17%, among those with 5-year-olds, these rates are higher than 50% [2].

In addition to its high prevalence, ECC is a matter of concern because of the severe implications it may have on the quality of life and well-being of children and their families. Children with ECC experience impairment of different dimensions in their life. The negative impact can range from difficulty performing daily activities such as eating and sleeping [5, 6] to problems in their growth and development, pain and the need for hospitalizations or emergency room visits [2]. This negative impact may be minimized by the dental treatment under general anesthesia though there is associated morbidity [7], sedation [8] or non-pharmacologic techniques for behavior management [9].

Although scientific evidence [7–9] shows that ECC treatment has definite and essential outcomes for improving the child and parents’ quality of life, approximately 621 million children worldwide have untreated cavitated lesions [1]. This alarming data on dental caries and its non-treatment may reveal that low priority is given to children under 6 years of age and the failures in the primary and secondary preventive measures [10]. Also, it should be emphasized that the treatment is technically challenging, and is often dictated by the extent of the lesions, child behavior [11] and the costs for patients/reimbursement system for dentists [12]. The high prevalence of the disease, added to its impact at both individual and collective levels, the possibility of prevention and treatment performed ECC a public health problem [13].

The decision to treat the ECC should be based on individual (such as risk assessment) and family (e.g., compliance of the patient’s caregiver’s willingness to change behaviors that affect oral health) [12, 14], as well as the professional’s experience [11]. As an aid for the decision-making process, there are several published guidelines/guidance/policies. However, considering that ECC is a problem that affects children worldwide, it is essential to know if the recommendations are similar among different countries and over time. This study intends to provide a summary of the guidelines/guidance/policies and a critical analysis of the available information. The aim of this review is to search for scientific evidence of the following question: what are the recommended procedures for the management of ECC lesions?

## Methods

This scoping review was developed following the recommendations of Arksey and O’Malley’s [15] and Joanna Briggs Institute [16] and reported according to the Preferred Reporting Items for Systematic reviews and Meta-Analyses extension for Scoping Reviews) (PRISMA-ScR) [17] (Additional file 1). The protocol was registered with the Open Science Framework on 28 January 2019 (https://osf.io/5xh2j). This type of review is indicated to synthesize or group existing evidence on a broad, complex or heterogeneous theme. For this, the following steps are recommended: research question formulation (broad or topic-focused questions), searches of references and their selection according to inclusion and exclusion criteria, extraction and presentation of results, critical evaluation (optional), synthesis of results and consultation (optional). Risk of bias assessment is not applicable for this review [16].

We included guidelines/guidance/policies that recommended procedures for the management of ECC lesions. According to National Institute for Health and Care Excellence (NICE) [18], guidelines involved the evidence-based recommendations; guidance makes evidence-based recommendations developed by independent committees and consulted on by stakeholders; policies are statements relating to institutions positions on various health issues [18]. Language, date of publication and publication status had no restrictions. Observational studies (cross-sectional, case-control and cohort), case reports, interventional studies (clinical trials), reviews and documents (guidelines/guidance/policies) about prevention of dental caries were excluded.

To identify the studies, the following electronic databases were searched in February 2019: PubMed, Scopus, The Cochrane Library, Latin American and Caribbean Health Sciences Literature (Lilacs), Embase and The International Guideline Library. The search strategy included MeSH terms, synonymous, related terms and free terms related to preschool children and dental caries (Table 1). This search strategy was adapted for each electronic database. Furthermore, pediatric dentistry associations around the world were contacted by email or respective website was consulted to identify unpublished guidelines. Duplicated studies were excluded using a bibliographic citation management software (EndNote X7, Thomson Reuters, New York, USA).

**Table 1:** Search strategy for each electronic database

Eletronic database	Search strategy
PubMed	((((Child, Preschool [mh] OR Child Preschool [tiab] OR Pediatric [tiab] OR Paediatric [tiab] OR Infant [mh] OR Infant* [tiab]) AND (Dental care [mh] OR Dental care [tiab] OR Dental treatment [tiab]) AND (Dental Caries [mh] OR Dental Decay [tiab] OR Dentins Carious [tiab] OR White Spots [tiab] OR White Spot [tiab] OR White Spot Dental [tiab] OR Early childhood caries [tiab] OR ECC [tiab] OR Tooth decay [tiab] OR Carious lesion [tiab])))) AND ((((Clinical pathway[mh] OR Clinical protocol[mh] OR Consensus[mh] OR Consensus development conferences as topic[mh] OR Critical pathways[mh] OR Guidelines as topic [Mesh:NoExp] OR Practice guidelines as topic[mh] OR Health planning guidelines[mh] OR guideline[pt] OR practice guideline[pt] OR consensus development conference[pt] OR consensus development conference, NIH[pt] OR position statement*[tiab] OR policy statement*[tiab] OR practice parameter*[tiab] OR best practice*[tiab] OR standards[ti] OR guideline[ti] OR guidelines[ti] OR ((practice[tiab] OR treatment*[tiab]) AND guideline*[tiab]) OR CPG[tiab] OR CPGs[tiab] OR consensus*[tiab] OR ((critical[tiab] OR clinical[tiab] OR practice[tiab]) AND (path[tiab] OR paths[tiab] OR pathway[tiab] OR pathways[tiab] OR protocol*[tiab])) OR recommendat*[ti] OR (care[tiab] AND (standard[tiab] OR path[tiab] OR paths[tiab] OR pathway[tiab] OR pathways[tiab] OR map[tiab] OR maps[tiab] OR plan[tiab] OR plans[tiab])) OR (algorithm*[tiab] AND (screening[tiab] OR examination[tiab] OR test[tiab] OR tested[tiab] OR testing[tiab] OR assessment*[tiab] OR diagnosis[tiab] OR diagnoses[tiab] OR diagnosed[tiab] OR diagnosing[tiab])) OR (algorithm*[tiab] AND (pharmacotherap*[tiab] OR chemotherap*[tiab] OR chemotreatment*[tiab] OR therap*[tiab] OR treatment*[tiab] OR intervention*[tiab]))))
Scopus	#1 TITLE-ABS-KEY (“Child Preschool”) OR TITLE-ABS-KEY (Pediatric) OR TITLE-ABS-KEY (Paediatric) OR TITLE-ABS-KEY (Infant*) #2 TITLE-ABS-KEY (“Dental care”) OR TITLE-ABS-KEY (“Dental treatment”) #3 TITLE-ABS-KEY (“Dental Caries”) OR TITLE-ABS-KEY (“Dental Decay”) OR TITLE-ABS-KEY (“Dentins Carious”) OR TITLE-ABS-KEY (“White Spots”) OR TITLE-ABS-KEY (“White Spot”) OR TITLE-ABS-KEY (“White Spot Dental”) OR TITLE-ABS-KEY (“Early childhood caries”) OR TITLE-ABS-KEY (ECC) OR TITLE-ABS-KEY (“Tooth decay”) OR TITLE-ABS-KEY (“Carious lesion”) #4 TITLE-ABS-KEY (“Clinical pathway”) OR TITLE-ABS-KEY (“Clinical protocol”) OR TITLE-ABS-KEY (Consensus) OR TITLE-ABS-KEY (“Consensus development conferences as topic”) OR TITLE-ABS-KEY (“Critical pathways”) OR TITLE-ABS-KEY (“Guidelines as topic”) OR TITLE-ABS-KEY (“Practice guidelines as topic”) OR TITLE-ABS-KEY (“Health planning guidelines”) OR TITLE-ABS-KEY (guideline) OR TITLE-ABS-KEY (“practice guideline”) OR TITLE-ABS-KEY (“consensus development conference”) OR TITLE-ABS-KEY (“consensus development conference, NIH”) OR TITLE-ABS-KEY (“position statement*”) OR TITLE-ABS-KEY (“policy statement*”) OR TITLE-ABS-KEY (“practice parameter*”) OR TITLE-ABS-KEY (“best practice*”) OR TITLE-ABS-KEY (standards) OR TITLE-ABS-KEY (guideline) OR TITLE-ABS-KEY (guidelines) OR ((TITLE-ABS-KEY (practice) OR TITLE-ABS-KEY (treatment*)) AND TITLE-ABS-KEY (guideline*)) OR TITLE-ABS-KEY (CPG) OR TITLE-ABS-KEY (CPGs) OR TITLE-ABS-KEY (consensus*) OR ((TITLE-ABS-KEY (critical) OR TITLE-ABS-KEY (clinical) OR TITLE-ABS-KEY (practice)) AND (TITLE-ABS-KEY (path) OR TITLE-ABS-KEY (paths) OR TITLE-ABS-KEY (pathway) OR TITLE-ABS-KEY (pathways) OR TITLE-ABS-KEY (protocol*))) OR TITLE-ABS-KEY (recommendat*) OR (TITLE-ABS-KEY (care) AND (TITLE-ABS-KEY (standard) OR TITLE-ABS-KEY (path) OR TITLE-ABS-KEY (paths) OR TITLE-ABS-KEY (pathway) OR TITLE-ABS-KEY (pathways) OR TITLE-ABS-KEY (map) OR TITLE-ABS-KEY (maps) OR TITLE-ABS-KEY (plan) OR TITLE-ABS-KEY (plans))) OR (TITLE-ABS-KEY (algorithm*) AND (TITLE-ABS-KEY (screening) OR TITLE-ABS-KEY (examination) OR TITLE-ABS-KEY (test) OR TITLE-ABS-KEY (tested) OR TITLE-ABS-KEY (testing) OR TITLE-ABS-KEY (assessment*) OR TITLE-ABS-KEY (diagnosis) OR TITLE-ABS-KEY (diagnoses) OR TITLE-ABS-KEY (diagnosed) OR TITLE-ABS-KEY (diagnosing))) OR (TITLE-ABS-KEY (algorithm*) AND (TITLE-ABS-KEY (pharmacotherap*) OR TITLE-ABS-KEY (chemotherap*) OR TITLE-ABS-KEY (chemotreatment*) OR TITLE-ABS-KEY (therap*) OR TITLE-ABS-KEY (treatment*) OR TITLE-ABS-KEY (intervention*))) #5 TITLE-ABS-KEY (guideline*) OR TITLE-ABS-KEY (practice guideline) OR TITLE-ABS-KEY (Clinical Practice Guideline) OR TITLE-ABS-KEY (recommendat*)
Cochrane Library	#1 (MeSH descriptor: [Child, Preschool] explode all trees) OR ((Child Preschool):ti,ab,kw) OR ((Pediatric):ti,ab,kw) OR ((Paediatric):ti,ab,kw) OR (MeSH descriptor: [Infant] explode all trees) OR ((Infant*):ti,ab,kw) #2 (MeSH descriptor: [Dental Care] explode all trees) OR ((“dental care”):ti,ab,kw) OR ((“Dental treatment”):ti,ab,kw) #3 (MeSH descriptor: [Dental Caries] explode all trees) OR ((“Dental Decay”):ti,ab,kw) OR ((“Dentins Carious”):ti,ab,kw) OR ((“White Spots”):ti,ab,kw) OR ((“White Spot”):ti,ab,kw) OR ((“White Spot Dental”):ti,ab,kw) OR ((“Early childhood caries”):ti,ab,kw) OR ((ECC):ti,ab,kw) OR ((“Tooth decay”):ti,ab,kw) OR ((“Carious lesion”):ti,ab,kw)
Lilacs	dental caries [Words] and guideline [Words]
Embase	((‘dental caries’/exp. OR caries:ab,ti) AND (‘protocol’/exp. OR ‘consensus’/exp. OR ‘guideline’/exp. OR ‘policy’/exp)) AND (‘preschool child’/exp. OR child:ab,ti OR ‘pediatrics’/exp)
The International Guidelines Library	Carie*

The selection of articles was carried independently by two calibrated researchers (KAV and PCF). First of all, the reviewers screened the titles and abstracts of the studies retrieved. Next, to confirm the inclusion, they read the full text of the studies considered potentially eligible in the screening step. Disagreements were solved by consensus.

Data of the included studies were extracted using a standardized data collection form designed for this scoping review. The following data were extracted: authors or association, year, location and recommended procedures management for ECC lesions. A summary of the results was provided in the text and presented in tables. The documents replaced by other updated were presented as Additional file 2.

## Results

### Study selection

**Fig. 1:**
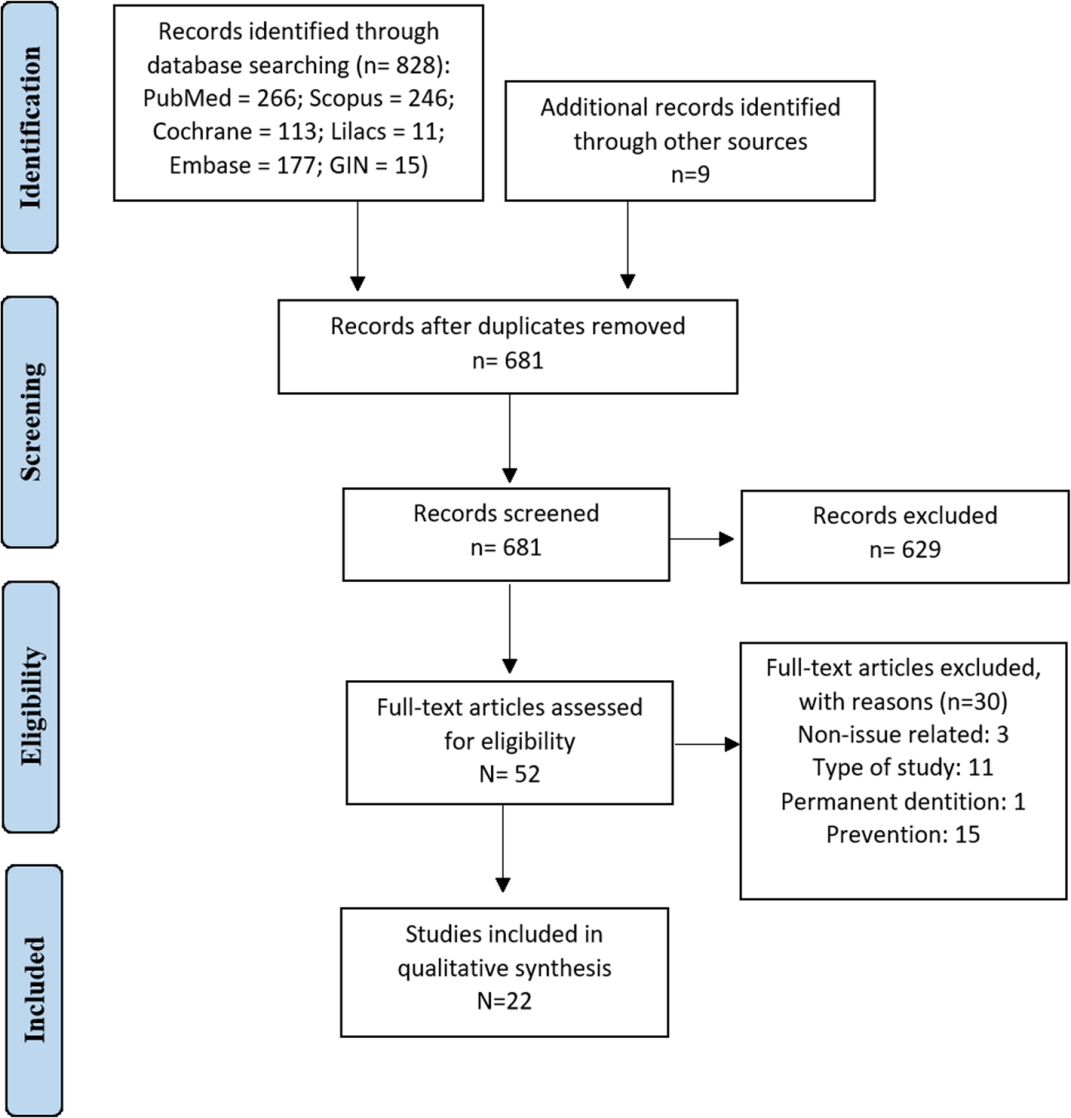
Flow diagram of literature search

A total of 837 documents were identified in the electronic (database) and manual (email send to Pediatric Dentistry Associations and search in these websites) search. After removing the duplicated studies, 681 remained. Among them, 629 were excluded based on the title and abstract, due to the following reasons: non-issue related (*n* = 595) and type of study (*n* = 34). Of the 52 full-text studies assessed for eligibility, 22 remained and were included in this scoping review (Fig. 1). Seven were policies, one was a guidance and 14 were guidelines (Tables 2 and Additional file 2). Of the 22 documents inserted, nine were replaced by updated ones and presented in Additional file 2. The final 13 updated documents are presented in Table 2.

**Table 2:** Characteristics of selected studies and recommended procedures for ECC management

Author, year	Country and/or Association	Recommended procedures management
**Policies**		
Asociación Argentina de Odontología para Niños [19]	Argentina	Remineralization with fluorides: systemic fluoride, topical fluoride- including fluoride toothpaste [6 months to 2 years: do not use; < 500 ppm for young children; 1000–1450 for children> 6 years]). Management of cavitated lesions: atraumatic restorative treatment, restoration with glass ionomer cement, composite resins and amalgam, steel crowns on primary molars
Kandiah et al., 2010 (replace Rayner et al., 2003 [20]; Fayle et al., 2001 [21]) [22]	British Society of Paediatric Dentistry (United Kingdom)	Management of active dental caries requires a combination of: - Prevention: water fluoridation, use of fluoride toothpaste and professional topical fluoride application, diet counselling, and provision of fissure sealants; -Restoration: use of stainless-steel crowns, and plastic restorations on small one and two surface cavities; - Pulp management, if necessary; -extraction. * Inhalation sedation and general anesthesia (GA) should be available for anxious children. GA can be used in cases of extensive disease.
American Academy of Pediatric Dentistry, 2016 [23] (replace AAPD, 2008 [24])	American Academy of Pediatric Dentistry (United States)	Anticariogenic agents (fluoride toothpaste and fluoride varnish) Definitive restorative Interim therapeutic restorations (ITR) or silver diamine fluoride (young children) Stainless steel crowns (advanced cases) *The selection of treatment is determined by the extend of the disease process, the patient’s developmental level and the comprehension skills. *More emphasis on prevention and arrestment to minimize the necessity of use of sedation and general anesthesia.
Kuhnisch et al., 2016 [25]	European Academy of Paediatric Dentistry	Arrest caries: fluorides Non-cavitated caries lesions: non-invasively management in the majority of cases (diet, oral hygiene, fluoride use and sealing techniques). Persistent active lesions: non-cavitated lesions may be sealed; Cavitated lesions should be restored after excavate soft and wet dentine Pulp management or extraction, if necessary * Minimally invasive methods and procedures to reduce the need of extensive operative measures with sedation or general anesthesia, especially in young children.
**Guidance**		
Scottish Dental Clinical Effectiveness Programme, 2018 [26]	Scottish Dental Clinical Effectiveness Programme (Scotland)	Site-specific prevention (fluoride varnish, dietary advice, brush lesion) for initial lesion (outer third dentine) or arrested caries in all teeth, and for tooth near to exfoliation. Fissure sealants/infiltration for initial lesion in molar. Caries removal and restoration using Atraumatic Restorative Treatment (ART) approach, composite, resin modified glass ionomer, compomer or glass ionomer for advanced lesion in all teeth. Hall technique for advanced lesion in molar (specially for proximal lesions, but either for occlusal lesion if child is not cooperative enough for a good adhesive restoration). Non-restorative cavity control for tooth near to exfoliation, any tooth with arrested caries, advanced lesion in anterior tooth, advanced lesion in molars with extensive cavitation, and for unrestorable tooth (pain/infection free). Teeth pulpally involved: extraction or endodontic treatment. * Sedation and general anaesthesia can be considered for child who is pre-cooperative or unable to co-operate or who has multiple affected teeth.
**Guidelines**		
American Academy of Pediatric Dentistry, 2017 [27]	American Academy of Pediatric Dentistry (United States)	Use of 38% SDF for the arrest of cavitated caries lesions
American Academy of Pediatric Dentistry, 2016 [28]	American Academy of Pediatric Dentistry (United States)	Active surveillance in cases of incipient lesions ITR - until a time when traditional cavity preparation and restoration is possible *More emphasis on prevention and arrestment to minimize the necessity of use of sedation and general anesthesia.
American Academy of Pediatric Dentistry, 2016 [29] (replace AAPD, 2008 [30] and AAPD, 2004 [31])	American Academy of Pediatric Dentistry (United States)	Active surveillance Sealants for already exhibit incipient, non-cavitated carious lesions Restoration with glass ionomer cement or resin-based composites or amalgam ITR ART Preformed metal crowns – Hall technique Pre-veneered stainless steel crowns
American Academy of Pediatric Dentistry, 2014 [32] (replace AAPD, 2013 [33] and AAPD, 2010 [34])	American Academy of Pediatric Dentistry (United States)	1–2 years old: Restore cavitated lesions with ITR or definitive restorations; active surveillance for incipient lesions; > 3 years: Restoration of cavitated or enlarging lesions; incipient lesions - active surveillance, except for children with high risk and parent not engaged, in which cases incipient lesions should be restored. * Fluoridated toothpaste was recommended for all children
Brazilian Association of Pediatric Dentistry, 2014 [35] (replaced Reference Manual, 2009 [36])	Brazilian Association of Pediatric Dentistry (Brazil)	Non-invasive approach (fluoride, dietary advice, biofilm control) for active enamel lesions. Sealants for incipient lesions Dentin lesions: ART, restoration with resin, glass ionomer cement or modified resin glass ionomer
Ministerio de Salud, Gobierno de Chile [37] (replaced Ministerio de Salud, Gobierno de Chile, 2008 [38])	Ministerio de Salud, Gobierno de Chile (Chile)	Application of fluorides (varnish, gel, mouthwash, fluoridated toothpaste) Sealants for non-cavitated lesions Dentin lesions: ART, restoration with resin or glass ionomer cement, preformed crowns. Pulpal therapy
Uribe, 2006 [39]	Scottish Intercollegiate Guideline Network (Scotland)	Caries progressing into dentine: managed with a preventive (not specified), or a preventive and restorative approach. Indirect pulp capping: if complete caries removal is not possible Cavitated lesions- ART, restoration with amalgam, composite, resin-modified glass-ionomers, compomer or preformed metal crowns
Peariasamy et al., 2012 [40]	Malaysia	Non-cavitated proximal enamel lesions: resin infiltration system used in conjunction with fluoride. Teeth that require temporization: use of spoon excavators followed by sealing the teeth with glass ionomer cement. Restorative treatment with amalgam, composite, glass ionomer cement, resin modified glass ionomer, high-viscosity glass ionomer, polyacid modified composite resin, stainless steel crown. Teeth pulpally involved: extraction or endodontic treatment, based on patient’s cooperation, medical condition, infection, restorability, extent of caries, potential for malocclusion. * The use of general anesthesia may be considered for uncooperative children or children that require extensive treatment.

The websites of Pediatric Dentistry Associations were consulted, and emails requesting information about guideline or recommendations for treating carious lesions were sent to representatives of associations from 68 countries. All continents have been contemplated. Of this total of emails sent, only eight responded that they did not have a country-specific guideline (Belgium, Sweden, Kenya, South Africa) or adopted those suggested by American Academy of Pediatric Dentistry and European Academy of Paediatric Dentistry (Serbia, Philippines, Mexico, Croatian). Guidelines of the United States, Brazil, Malaysia, United Kingdom were available on the websites. The document prepared in Brazil was translated and adopted by some Latin American countries, such as Paraguay. A more current version of Chile’s guideline was available on that country’s Ministry of Health website. Documents of Academy of Paediatric Dentistry (United States), European Academy of Paediatric Dentistry (United Kingdom) and Chilean Ministry of Health also where be identified in the electronic databases search.

Considering all the documents, most of the studies were published in English between 2011 and 2018. Documents were identified from the United States, United Kingdom, Malaysia, Brazil, Chile, Argentina, the British Society of Paediatric Dentistry and the European Academy of Paediatric Dentistry.

**Fig. 2:**
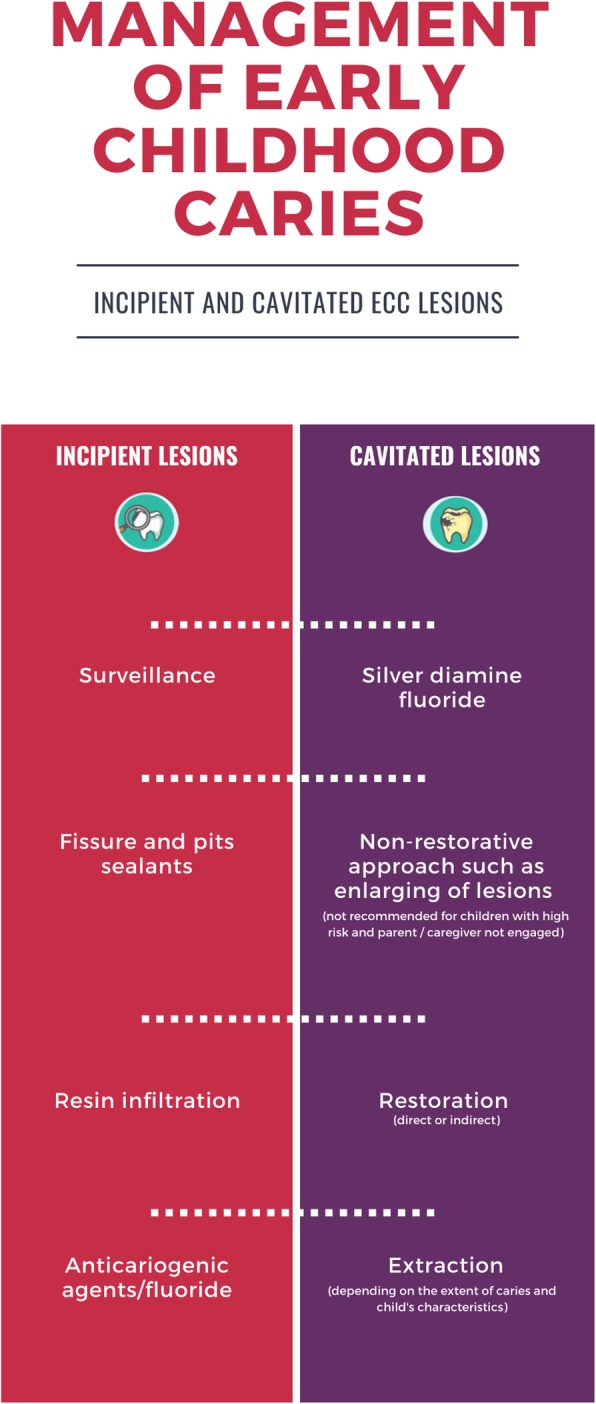
Procedures for the management of ECC lesions

Different procedures were suggested for the management of ECC lesions, ranging from surveillance to extraction (Fig. 2). Active surveillance – careful monitoring of lesions progression and application of prevention measures - was recommended for incipient lesions, except for children older than 3 years with high caries risk and whose parents were not engaged [28, 29, 32]. For incipient lesions, other options were fissure and pit sealants [25, 26, 3738], resin infiltration for proximal enamel lesions [26, 40], the use of anti-cariogenic agents [30] (Additional file 2) and fluoride [19, 23, 25, 26, 36, 37, 40]. Home-based fluoride was recommended by just eight studies [19, 21, 23, 32, 37]. For the arrest of cavitated caries lesions, the use of 38% silver diamine fluoride (SDF) was recommended [27], especially for young children [23], just for American Academy and just after 2016.

Almost all documents advocated the restoration of primary teeth with cavitated carious lesion [22, 23, 25, 26, 29, 38–40]. Interim therapeutic restorations (ITR) may be indicated for caries control in children with a great deal of cavitated carious lesion, as well as for the uncooperative patient, small children and patients with special health care needs that could not receive permanent restorations [23, 28, 29, 32]. To restore primary teeth, it was recommended the use of different materials/techniques, such as Atraumatic Restorative Treatment - ART [19, 29, 37–39]; restoration with glass ionomer cement [19, 26, 29, 37–39] or resin-based composites [19, 21, 26, 29, 37–40] or amalgam [19, 29, 37, 39, 40]; stainless steel crowns [19, 23, 26, 29, 37, 39, 40]. The Hall Technique was first added at 2013 [37]. The provision of prosthetic restoration, fixed or removable, was endorsed in children who lost teeth ([20, 30, 31] Additional file 2).

Extraction was also indicated [25, 26, 40], especially for teeth pulpal involved depending on the patient’s cooperation, medical condition, infection, the extent of caries and potential for malocclusion [40]. No other differences besides of that mentioned above (SDF and Hall Technique) were found among recommendations along time and in different places.

The use of advanced behavior guidance techniques to treat children with ECC was considered in six documents [23–26, 28, 40].

## Discussion

This scoping review was performed to identify what procedures were recommended for the management of ECC lesions and to promote critical analysis of the information available on the guidelines/guidance/policies. Different interventions were suggested for the management of ECC lesions, from active surveillance to extraction, depending on the disease process and the patient’s risk of caries. In fact, it is well known that the clinical decision-making for caries management on children should be based on the patient’s risk levels, current oral health status, age, and the engagement of caregivers with preventive strategies [13, 14].

Active surveillance was proposed by few recommendations, and just for incipient lesions. However, considering that the surveillance is a pivotal component of caries management, it should be recommended for all patients, aiming to both arrest lesions and monitor the progression [13]. For non-cavitated carious lesions, the use of professional fluoride (5% NaF varnish) associated with sealants on occlusal surfaces or with resin infiltration on approximal surfaces increase the chance of arresting or reversing lesions compared with no treatment [41], so whenever possible, these procedures should be performed. One cannot fail to mention, however, that for the resin infiltration, to our knowledge, just a single commercial product holds the patent so that this fact can restrict its use. When it was not available, professionals can use other approaches, such as fluoride toothpaste and dental floss, and/or fluoride varnish, although these conventional management are less effective, probably because they demand a high level of patient compliance [42].

The use of fluoride toothpaste has been recommended in a few documents [22, 23, 32], although it is easy to apply and is currently recommended as a prevention measure secondary to ECC. Only from the document published in Argentina, the recommended fluoride concentration and amount of toothpaste for each age were addressed [19]. However, the recommendations are not out of date. This information has recently been discussed, and it is recommended that for all children a concentration of at least 1000 ppm fluoride should be used for twice-daily brushing, following the recommended amount of toothpaste for each age [4]. For children under 3 years old, the amount of fluoridate toothpaste corresponding to smear size is recommended, while children under 3–6 years old should use the amount corresponding to pea-size [2].

Almost all documents recommended restoring cavitated lesions, which historically is the approach used to management of caries [13]. Nonetheless, it is recognized that although restorative approaches are possible for the management of ECC, low scientific evidence was found for this intervention in anterior teeth [43]. It is known that there is a variety of nonrestorative procedures that can be used to treat carious lesions both on anterior and posterior teeth [41]. Two studies demonstrated that keeping cavities in primary molars without biofilm might be a treatment option to arrest cavitated lesions [44, 45]. The success of this therapy - keep cavitated lesions without inserting restorative materials (non-restorative approach) - depends on the attitudes of patients and their families to brush the lesions and change habits [14, 46].

Although SDF has been used for more than four decades, it was recommended just in 2013 [37]. It can be explained by the fact that at the beginning, few countries used SDF. The number of studies about SDF published in English before 2009 is limited because before this year, SDF was commonly used just in Argentina, Australia, Brazil, China and Japan [47]. Nonetheless, SDF showed to be more effective than other interventions for control caries progression in primary teeth [48] as well as to prevent dental caries in the entire dentition [49]. Accordingly, one systematic review pointed out that there is a high level of evidence for the potential of the SDF for arrest carious lesion (cavitated and non-cavitated) [43]. This recommendation is confirmed in a document recently published by the World Health Organization [50]. Different restorative materials were recommended to primary teeth, based on the involvement of different surfaces. One systematic review found that there is no different among compomer, resin-modified glass ionomer cement, amalgam and composite resin, but conventional glass ionomer cement had a higher risk of failure in primary molars compared to other materials [51]. On the other hand, another systematic review found that composite resin, compomer, resin-modified glass ionomer cement, and glass ionomer cement showed similar results for the restoration of primary molars [52]. Although ART could be considered an alternative for restoring occlusal [53] and occlusoproximal [53, 54] cavities in primary molars, just six guidelines cited it. Bearing all these aspects in mind, the clinical decision-making to choose one material should consider besides the type of cavity, the patient’s/family’s wish, the professional’s ability, the child’s age, the child’s behavior and treatment’ costs and availability.

Considering that around 9% of pediatric patients presented dental fear/anxiety or dental behavior management problems [55], a portion of the child population may need the use of pharmacological behavior management techniques to perform the dental treatment, but just six documents considered this possibility [22, 23, 25, 26, 28, 40]. Sedation and general anesthesia aimed to provide safe and effective dental care [27, 56] and had proved to improve the child’s behavior in the dental chair along the time [57] and to improve the oral health-related quality-of-life [7], respectively.

Our scoping has limitations that involve the inclusion of only documents available electronically. We recognize that, in some countries, there may be guidelines/policies/guidance in printed books and manuals that were not considered in our study. To minimize this limitation, efforts were performed to request associations from different countries to send any documents regarding the management of ECC lesions.

Recently, a manual was developed and published by the World Health Organization [50]. This document is based on systematic reviews and WHO recommendations. The recommendations for arresting and restoration of carious lesions are similar to those observed in our study. This mainly included the use of sealants, fluoride and minimally invasive techniques for restoration such as ART. Our study advances by presenting information referring to guidelines, policies and guidance from different countries, allowing an overview of the management of ECC lesions. This scoping review brings a summary of the recommendations about the management of ECC lesions, which seems not to be previously described. This broad vision of what the recommendations around the world bring can help during the development/updating of guidelines, as well as it can help the dental team critically analyze guidelines before implementing procedures. Unfortunately, although all efforts were made to find all recommendations, it cannot be ruled out that some of them were not found. Based on our results, it can be concluded that there is no difference among recommendations from different places and from different years about the management of dental caries. Similarly, to the conclusions of a previous systematic review, we endorsed the use of SDF for cavitated or non-cavitated lesions, the use of fluoride varnish for non-cavitated lesions, and a cautious indication of restorative approaches, especially for anterior teeth [43].

## Conclusions

ECC management involves analyzing the extent of caries lesions and children’s characteristics, such as their behavior. The documents reviewed are similar in their recommendations; the most indicated procedures were non-operative (such as active surveillance, the use of professional fluoride associated with sealants on occlusal surfaces or with resin infiltration on approximal for arresting or reversing lesions), restorative and extraction.

## Supplementary information

**Additional file 1.** Preferred Reporting Items for Systematic reviews and Meta-Analyses extension for Scoping Reviews (PRISMA-ScR) Checklist

**Additional file 2.** Characteristics of replaced studies and recommended procedures for ECC management.

## Data Availability

The data from this scoping review may be made available by the corresponding author through the email.

## Abbreviations

ART: Atraumatic restorative treatment
ECC: Early childhood caries
ITR: Interim therapeutic restorations
PRISMA-ScR: Preferred Reporting Items for Systematic reviews and Meta-Analyses extension for Scoping Reviews
MeSH: Medical Subject Headings
NICE: National Institute for Health and Care Excellence
SDF: Silver diamine fluoride
