# Supplementary material for: Recommended procedures for the management of early childhood caries lesions – a scoping review by the Children Experiencing Dental Anxiety: Collaboration on Research and Education (CEDACORE)
Source: BMC Oral Health. 2020 Mar 17;20:75. doi: 10.1186/s12903-020-01067-w (PMC7079355; doi:10.1186/s12903-020-01067-w)
Supplement: Supplementary file 2 — Additional file 2. Characteristics of replaced studies and recommended procedures for ECC management. [file 12903_2020_1067_MOESM2_ESM.pdf]

Additional File 2. Characteristics of replaced studies and recommended procedures for ECC management

Author, year	Country and/or association	Recommended procedures for management
**Policies**		
Rayner et al., 2003 [21]	British Society of Paediatric Dentistry (United Kingdom)	Interim therapeutic restorations<br>Permanent restorations<br>Prosthetic restoration<br>Extraction<br>* Treatment under general anesthesia should be considered.
Fayle et al., 2001 [22]	British Society of Paediatric Dentistry (United Kingdom)	Active dental caries:<br>- preventive strategies (water fluoridation, fluoride in toothpaste and supplements, dietary counseling, fissure sealants, oral hygiene instruction, professional topical application of fluoride) alone are rarely adequate;<br>- restorations with amalgam, composite resins, compomers or glass ionomer cements for one or two surface restorations, or stainless-steel crowns for extensive and/or multi-surface caries;<br>- pulp treatment, if necessary;<br>- extraction under local or general anesthesia.

		* Oral or inhalation conscious sedation can be used as an essential adjunct. General anesthesia can be indicated in cases of multiple extractions and/or restorations in young or anxious children.
American Academy of Pediatric Dentistry, 2008b [24]	American Academy of Pediatric Dentistry (United States)	Anticariogenic agents (not specified)<br>ITR<br>Stainless-steel crowns<br>* To perform treatment effectively and efficiently, the practitioner often must use advanced behavior guidance techniques (protective stabilization and/or sedation or general anesthesia).
**Guidelines**		
Ministerio de Salud, Gobierno de Chile, 2008 [38])	Ministerio de Salud, Gobierno de Chile (Chile)	Application of fluorides (varnish, gel, mouthwash, fluoridated toothpaste)<br>Sealants for non-cavitated lesions<br>Dentin lesions: ART, restoration with resin or glass ionomer cement, preformed crowns.<br>Pulpal therapy
American Academy of Pediatric Dentistry, 2008a [30] (replaced	American Academy of Pediatric Dentistry (United States)	Sealants for incipient lesions<br>ITR<br>ART<br>Restoration with glass ionomer cement or resin-based composites or amalgam

AAPD, 2004 [31])		Stainless-steel crowns<br>Fixed prosthetic restoration<br>Removable prosthetic appliances
American Academy of Pediatric Dentistry, 2004 [31]	American Academy of Pediatric Dentistry (United States)	Sealants for incipient lesions<br>ITR<br>ART<br>Restoration with glass ionomer cement or resin-based composites or amalgam<br>Stainless-steel crowns<br>Fixed prosthetic restoration<br>Removable prosthetic appliances
American Academy of Pediatric Dentistry, 2013 [33] (replaced AAPD, 2010 [34])	American Academy of Pediatric Dentistry (United States)	One to two years old: restore cavitated lesions with ITR or definitive restorations; active surveillance for incipient lesions<br>> Three years: restoration of cavitated or enlarging lesions; incipient lesions: active surveillance, except for children with high risk and parent not engaged, in which cases incipient lesions should be restored.<br>* Fluoridated toothpaste was recommended for all children, except for those less than two years old with low risk for caries.

American Academy of Pediatric Dentistry, 2010 [34]	American Academy of Pediatric Dentistry (United States)	One to two years old: restore cavitated lesions with ITR or definitive restorations; active surveillance for incipient lesions<br>> Three years: restoration of cavitated or enlarging lesions; incipient lesions: active surveillance, except for children with high risk and parent not engaged, in which cases incipient lesions should be restored<br>* Fluoridated toothpaste was recommended for all children.
Brazilian Association of Pediatric Dentistry, 2009 [35]	Brazilian Association of Pediatric Dentistry (Brazil)	Sealants for incipient lesions<br>ART<br>Restoration with resin, glass ionomer cement, amalgam, or stainless-steel crowns
